# Decline in Uptake of Childhood Vaccinations in a Tertiary Hospital in Northern Ghana during the COVID-19 Pandemic

**DOI:** 10.1155/2021/6995096

**Published:** 2021-12-14

**Authors:** Kingsley Appiah Bimpong, Benjamin Demah Nuertey, Anwar Sadat Seidu, Stephanie Ajinkpang, Alhassan Abdul-Mumin

**Affiliations:** ^1^Department of Pediatrics and Child Health, Tamale Teaching Hospital, Tamale, Ghana; ^2^Department of Public Health, Tamale Teaching Hospital, Tamale, Ghana; ^3^Department of Community Health, University of Ghana Medical School, Accra, Ghana; ^4^Department of Surgery, Tamale Teaching Hospital, Tamale, Ghana; ^5^Department of Pediatrics and Child Health, School of Medicine, University for Development Studies, Tamale, Ghana

## Abstract

At the beginning of the COVID-19 pandemic, early modelling studies estimated a reduction in childhood vaccinations in low- and middle-income countries. Regular provision of both curative and preventive services such as antenatal care and childhood immunizations has been negatively affected since the onset of the pandemic. Our study was aimed at examining the impact that the pandemic had on childhood vaccination services at the Tamale Teaching Hospital (TTH). A mixed methods study design was employed for the study, which was conducted at the Child Welfare Clinic (CWC) of the TTH. With quantitative approach, we retrospectively looked at the uptake of the various vaccines during the pandemic era, defined as the period between 1^st^ March 2020 and 28^th^ February, 2021, and the prepandemic era defined as the period 1^st^ March 2019 to 29^th^ February, 2020. The qualitative approach was used to understand the perspective of five healthcare providers at the CWC and the four caregivers of children who have missed a vaccine or delayed in coming, on the factors accounting for any observed change. Data analysis was done using Microsoft Excel 2016 and thematic content analysis. Quantitative data were presented in frequencies, percentages, and line graphs. With the exception of the Measles Rubella (MR) 2 vaccine, we observed a decline ranging from 47% (2298) to 10.5% (116), with the greatest decline seen in the BCG and the least decline seen in the MR1 vaccine. The month of May 2020 saw the greatest decline, that is, 70.6% (813). A decline of 38.3% (4473) was noted when comparison was made between the designated prepandemic and pandemic eras, for all the vaccines in our study. Fear of COVID-19 infection and misinformation were commonly given as reasons for the decline. Catch-up immunization schedule should be instituted to curtail possible future outbreaks of vaccine-preventable diseases.

## 1. Introduction

The World Health Organization (WHO) in January 2020 declared the COVID-19 a public health emergency of international concern, and subsequently, it was declared a pandemic in March 2020 [1]. Ghana has been one of the worse affected countries on the African continent [2].

Early modelling studies estimated an 18.5% to 51.9% reduction in early childhood vaccinations in low- and middle-income countries (LMICs) as a result of factors such as reduction in demand, supply, and access to vaccines [3]. Regular provision of both curative and preventive services (immunizations) such as antenatal care (ANC) and childhood immunizations has been negatively affected [4, 5]. Moyer et al. noted that in Ghana, the current pandemic was increasing anxiety among pregnant women, with some avoiding ANC service and reconsidering giving birth at home rather than in hospitals [6]. Also, Abdul-Mumin et al. reported a decline in admissions at our neonatal intensive care unit of the Tamale Teaching Hospital (TTH) during the current pandemic [7].

Abbas et al. however suggested that routine childhood vaccinations should continue as the risks of not sustaining it outweighs the excess deaths from COVID-19 that might result from attendance to clinics where vaccinations are carried out, in Africa [8]. Also, there is a risk of outbreaks of vaccine-preventable diseases, if attempts are not made to address the delay and miss in vaccinations [9].

The WHO has noted that measles vaccination for about 117 million children could be affected by measures taken by countries to avert the spread of the COVID-19 infection, with possible suspension of intended health campaigns [10]. Both developed and developing countries have not been spared this dangerous decline [11]. The Center for Disease Control (CDC) reported a decline in the ordering of pediatric vaccines and dose administrations [12, 13]. In England, it was shown that vaccinations for measles, mumps, and rubella decreased in 2020, even before the introduction of social distancing measures [14]. In Pakistan, a provincial study noted that one in every two children missed their routine vaccination, with a greater than 50% reduction in overall immunization coverage as a result of lockdown measures to curtail the spread of the virus, which could be explained by the reduced demand and supply of these vaccines [15].

In the previous Ebola disease outbreak that rocked parts of West Africa, it was noted that childhood vaccination services declined, with reports suggesting that a recovery to pre-Ebola levels was not noted even after the disease outbreaks [16]. This current pandemic has not been different with various studies showing decline in jurisdictions where studies have been carried out [9, 12, 13].

At the beginning of the first wave, a number of measures were taken in the TTH, which is a tertiary facility with a bed capacity of 860, serving the five (5) northern regions of Ghana. Although essential services including childhood vaccinations continued, measures such as suspension of regular specialist clinics and reduction in numbers of patients seen at the ANC were instituted. A formal assessment is hence warranted to document how these disruptions might have also affected vaccination services. The outcome of this study will help policymakers come out with catch-up vaccination schemes if the need be and also provide a guidance for future pandemics.

## 2. Methodology

### 2.1. Study Design and Area

A mixed (quantitative and qualitative) method was employed for the study, which was conducted at the Child Welfare Clinic (CWC) of the TTH. The hospital is located in Tamale, the regional capital of the Northern Region of Ghana. On average, 8000 deliveries are conducted in the hospital per annum. The hospital also hosts the only treatment center for severe COVID-19 cases in the catchment area. With the quantitative approach, the study team retrospectively looked at the uptake of the various vaccines during the pandemic era and the prepandemic era. The qualitative approach was done to understand the perspective of healthcare providers at the clinic and the caregivers of children who have missed a vaccine or delayed in coming, on the factors accounting for any observed change. The clinic provides childhood vaccination services to inborn and outborn babies. The TTH is the only tertiary hospital located in the northern part of Ghana.

### 2.2. Operational Definitions

*Pandemic period*: 1^st^ March 2020 to 28^th^ February, 2021^∗^

*Prepandemic period*: 1^st^ March 2019 to 29^th^ February, 2020^∗^

^∗^The data for December 2020 was missing because in December 2020, the facility migrated from one electronic medical record to another, which caused a temporary disruption in data collection for that month. Thus, we excluded the month of December for both the prepandemic and pandemic periods from the data analysis.

### 2.3. Study Population

The quantitative study included data from immunization coverage during our study period. Covered vaccinations were Bacillus Calmette-Guérin (BCG) at birth, which was used as a proxy for the vaccines taken at birth. Pentavalent 1 was used as a proxy for vaccines taken at 6 weeks, pentavalent 2 was used as a proxy for vaccines taken at 10 weeks, and pentavalent 3 was used as a proxy for vaccines taken at 14 weeks. Measles Rubella (MR1) was used as a proxy for vaccines taken at 9 months, and MR2 was used as a proxy for vaccines taken at 18 months [17].

For the routine immunization records, the age of child, vaccine, batch number of vaccine, and date of administration are documented for each child in a vaccine register and the child health record book is carried by the client. From the vaccination register, the community health nurses transfer the data into a tally book, which tallies administered vaccines, for example, pentavalent vaccine 1, pentavalent vaccine 2, and pentavalent vaccine 3 for pentavalent vaccine recommended for 6, 10, and 14 weeks, respectively, irrespective of the age at which it was administered provided it falls within the acceptable age range for the respective vaccines. In the vaccine register and the child health record book, it is possible to determine late doses or clients who defaulted. However, the tally register would give the vaccine administered only without segregating data into the age at which each antigen was administered.

The qualitative component of the study involved in-depth interviews and a focused group discussion. In-depth interviews were conducted with staff of the CWC in order to get their perspective on possible factors for any observed change in immunization services during the pandemic period. Also, focused group discussion was done with caregivers of children who missed out or delayed on any immunization schedule during the pandemic period. Six (6) staff were approached out of which five (5) agreed to participate in the study. Seven (7) caregivers were approached out of which four (4) consented to the study. These interviewees were selected by the first and fourth authors (KAB and SA). The first author (KAB) is a medical doctor, and the fourth author (SA) is a pediatric nurse specialist, both at the department of pediatrics and child health of the TTH. Staff at different grades, who were present at the Child Welfare Clinic before the pandemic and during the pandemic, were approached. This category of staff was approached to avoid bias, as they were involved in childhood vaccinations during our designated pandemic era and prepandemic era. Also, caregivers who presented for vaccination during our study period were inquired if they missed any vaccination. Those who missed a vaccination during our designated pandemic era were approached.

### 2.4. Data Collection Procedure and Tool

Data collection was done via two means. The study team used a structured data collection sheet attached as supplement 1 to collect data on the various vaccine doses administered during the study period. This information was obtained from the Public Health Department of the Hospital which keeps records of all immunization coverage in the hospital. This information was entered into a Microsoft Excel sheet and cleaned for analysis.

The other means for our data collection was in-depth interview and focused group discussion, using an interview guide, attached as supplement 2 and supplement 3 for the staff and caregivers, respectively. Perspective of some of the staff at the CWC was sought on any observed change during the pandemic period and possible factors accounting for this. Sessions took between seven and fifteen minutes. Also, some caregivers of children who delayed or missed any of the vaccination schedule were interviewed through a focused group discussion to understand reasons underlying why they missed or delayed the schedule. The focused group session lasted for sixteen minutes.

### 2.5. Data Analysis

Data analysis was done using Microsoft Excel 2016, Statistical Package for Social Sciences (SPSS) version 23, and thematic content analysis. Descriptive statistics of frequencies and percentages were used to assess the changes noted. Also, comparison of trends of the vaccination in the prepandemic and the pandemic era was done using line graphs. A Wilcoxon signed rank test was used to compare the frequency of monthly vaccinations for the two periods (prepandemic and pandemic). Statistical significance was determined at a *p* value less 0.05. Qualitative data were transcribed verbatim and checked for errors. Transcripts of the individual interviews were assigned series of codes and grouped into similar themes, using the thematic content analysis. These were supported with quotes.

### 2.6. Ethics Statement

Ethical clearance for this study was obtained from the ethical review committee of the TTH, with reference ID TTHERC/30/09/20/07. Written informed consent was obtained from all participants of the study. The first and fourth authors (KAB and SA) who conducted the interview explained the relevance of the study to the participants. Also, participants were made aware that participation was optional.

## 3. Results

### 3.1. Overall Vaccinations Done in the Prepandemic Era Compared to the Pandemic Era

The general change in each of the proxy vaccines studied is illustrated in Table 1. When we compared the prepandemic period to the pandemic period, we observed a general decline in all the vaccines included in the study, with the exception of the MR2 vaccine. This decline ranged from -47% to -10.5%, with the greatest decline seen in the BCG and the least decline seen in the MR1 vaccine. It was noticed that the decline improved with increasing age of vaccination.

### 3.2. Monthly Changes in Vaccines Administered

Monthly variations in the vaccines administered in the pandemic period were also compared with those in the prepandemic period. This is illustrated in Table 2. Ghana recorded its first case of COVID-19 in March 2020 [18]. It was observed that there was a sharp decline (45.6%) in the vaccination services in the following month. This decline peaked and worsened in May 2020 at 70.6% but continued throughout the study period, when the prepandemic vaccinations were compared with the pandemic vaccinations. A statistically significant *p* value of 0.003 was obtained when the monthly frequency of vaccination in the prepandemic period was compared with the pandemic period. The overall average decline in vaccinations was 38.3%, when we compared the prepandemic year to the pandemic year. With an estimated target population of 2293, the monthly percentage of vaccines administered was also obtained and is illustrated in Table 2.

### 3.3. BCG Doses Administered in the Prepandemic Era Compared to the Pandemic Era

**Figure 1:**
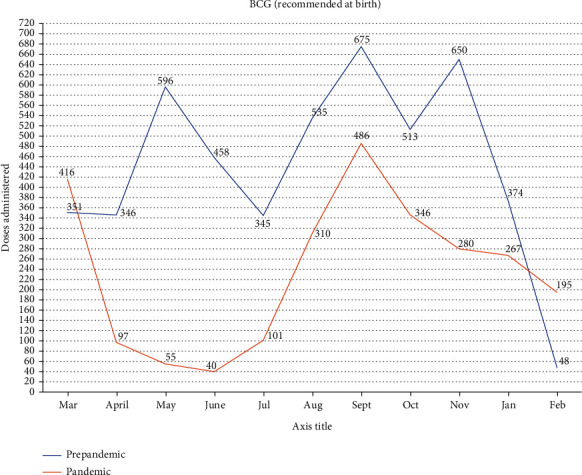
BCG doses administered in the prepandemic era compared to the pandemic era.

Throughout the study, BCG recorded on average the greatest decline of -47.0% as shown in Table 1. The monthly changes for the BCG vaccine are indicated in Figure 1. We recorded a sharp decline (-76.7%), when the April 2020 records were compared to the preceding month when the country recorded her index case. This decline continued until July, 2020, when figures begun to appreciate. However, the prepandemic doses administered were not reached in any of the months studied, until February, 2021.

### 3.4. PENTA 1 Doses Administered in the Prepandemic Era Compared to the Pandemic Era

**Figure 2:**
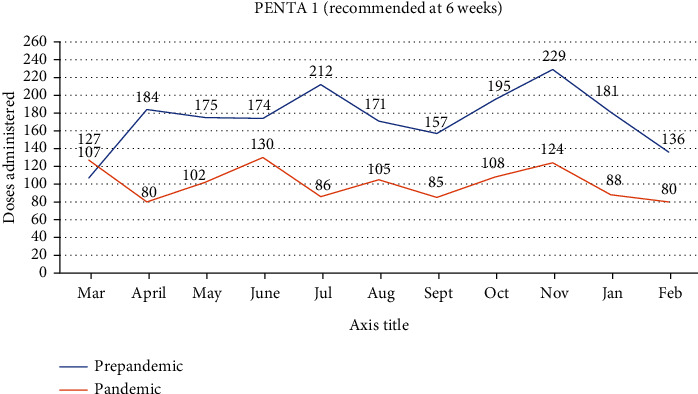
PENTA 1 doses administered in the prepandemic era compared to the pandemic era.

Again, we recorded a decline (-37.0%) in the vaccination coverage for PENTA 1, after Ghana recorded its index case, that is, when April 2020 was compared to March 2020, as shown in Figure 2. For PENTA 1, none of the prepandemic vaccination coverages was attained throughout the study period. Overall, an average of 42.0% decline was observed when the prepandemic doses administered were compared with the pandemic coverage. It is worth noting that, in the latter part (January and February, 2020) of our prepandemic period, the doses administered of these vaccines started declining as other countries started recording cases of the COVID-19.

### 3.5. PENTA 2 Doses Administered in the Prepandemic Era Compared to the Pandemic Era

**Figure 3:**
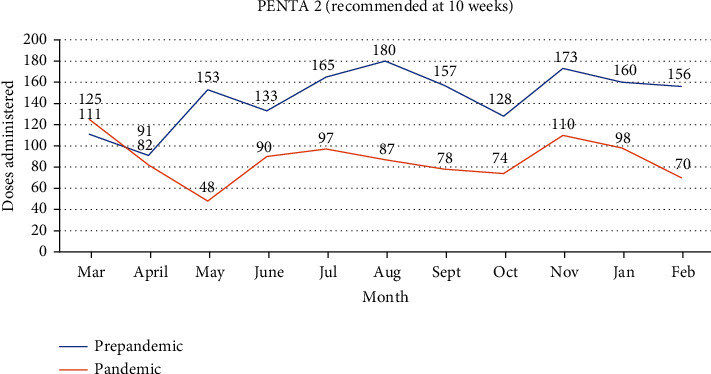
PENTA 2 doses administered in the prepandemic era compared to the pandemic era.

The trend for the change in PENTA 2 vaccination in the prepandemic period compared to the pandemic period is depicted in Figure 3. A drop of 34.4% was seen when the month of April 2020 was compared to March 2020, with a further 41.5 drop observed in May 2020. An overall average decline of 40.3% was seen in the pandemic period compared to the prepandemic period, as illustrated in Table 1.

### 3.6. PENTA 3 Doses Administered in the Prepandemic Era Compared to the Pandemic Era

**Figure 4:**
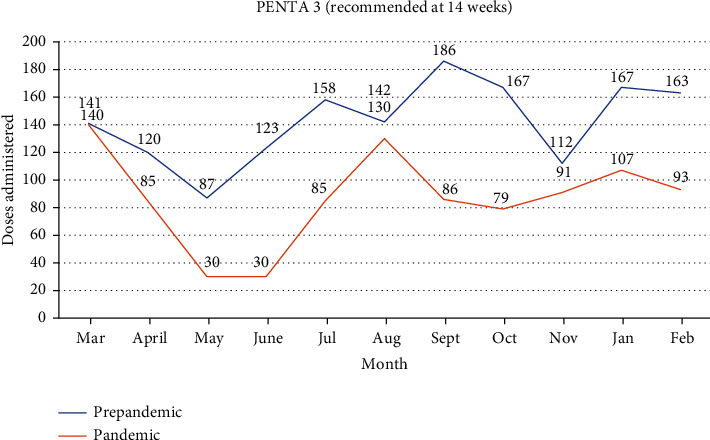
PENTA 3 doses administered in the prepandemic era compared to the pandemic era.

Figure 4 depicts the graphical changes observed in the PENTA 3 vaccine when the prepandemic era was compared to the pandemic era. A 39.3% drop in vaccination for PENTA 3 was seen in the first month after the index case was recorded in Ghana, with a further 64.7% drop seen in the following month. Prepandemic levels are also yet to be attained for this vaccine. In all the months considered, an average of 39.0% drop was observed when the prepandemic period was compared to the pandemic period, as shown in Table 1.

### 3.7. MR1 Doses Administered in the Prepandemic Era Compared to the Pandemic Era

**Figure 5:**
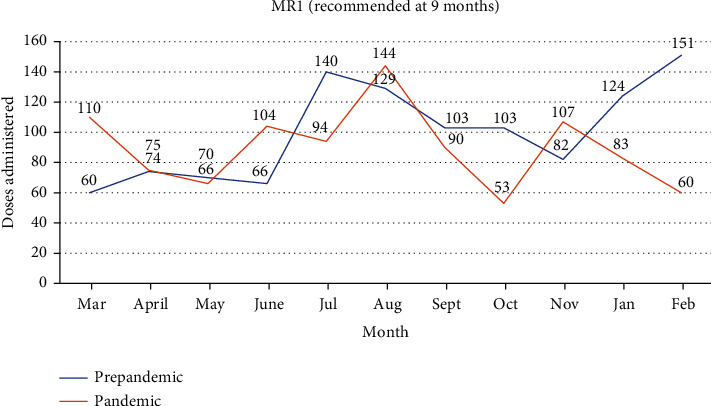
MR1 doses administered in the prepandemic era compared to the pandemic era.

The trend in change in the MR1 vaccine is shown in Figure 5. A 31.8% drop was noticed in the month after Ghana recorded its first case. A further 12% drop was recorded in the following month. However, coverage greater than the prepandemic levels was seen in some months (June, August, and November). An overall average decline of 10.5% was observed when the pandemic period was compared to the prepandemic periods.

### 3.8. MR2 Doses Administered in the Prepandemic Era Compared to the Pandemic Era

**Figure 6:**
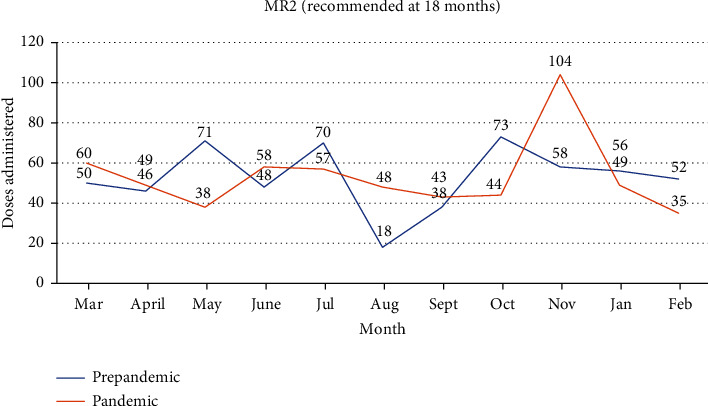
MR2 doses administered in the prepandemic era compared to the pandemic era. 7S.

Figure 6 shows the trend in change in MR2 vaccine, in the prepandemic period compared to the pandemic period. We recorded 15.9% drop in April 2020, compared to March 2020 when Ghana recorded its first case. A further 22.4% drop was seen in the month afterward. However, when all the numbers in the prepandemic period was compared to those in the pandemic period, the change that was noticed in this vaccine administration was insignificant (0.9%).

### 3.9. Response and Profile from Interviewees

The response rate for the staff of the CWC was 83%, and that of the caregivers was 57%. We interviewed staff who were working at the unit before and during the pandemic. Carefully, we approach staff of both higher and lower grades to ensure that our sample was representative. Caregivers interviewed were mothers of children who missed a vaccination schedule of their child during our designated pandemic period.

### 3.10. Staff's Perceived Change in Attendance at the CWC

All participants interviewed indicated that there was a reduction in attendance of mothers bringing their children for immunization, as indicated by one participant that “there was a great reduction in the number of women bringing their children for vaccination” and buttressed by another participant that “yes, there was a change, as the attendance reduced.”

### 3.11. Staff's Reasons for Observed Change

The fear of COVID-19 infection was one of the reasons perceived by staff to have resulted in the reduction of numbers seen at the child welfare clinic, during the pandemic period. Some caregivers were afraid of infecting themselves and their children, as they heard that the hospital was a treatment center and some staff may be infected by the virus too. These were buttressed by a respondent who stated that “the parents heard that we had a COVID-19 treatment center in the hospital, so some of them were afraid to even come near to the hospital.” Another participant also indicated that “the mothers were afraid of getting the infection in the hospital from staff.” “Some of the mothers thought some of the staff were having the COVID infection” was noted by another participant.

Misinformation from the general public is another reason accounting for the decline in attendance for the childhood immunizations. Caregivers were given information from the public that vaccination services have been halted as a result of the pandemic. This was illustrated by participants as “Because of the COVID, they said the vaccination was not going on.” Another participant also stated that mothers told them that “they heard that we were not giving the vaccination.” Again, the public was misinformed on the hospital's state as a treatment center. Although the teaching hospital had a treatment center, this was at a separate place, and clients coming for other services did not get in touch with the center.

It is worth noting that the demands of the pandemic meant that some staff had to be reassigned to the treatment centers. However, this did not have any impact on the vaccination services, as some staff were always available at working hours to attend to this. This was buttressed by one participant as: “Although some of the staff were re-assigned, it did not affect the clinic.”

### 3.12. Clients' Reasons for Missing or Delaying Vaccination Schedules

Fear is a major reason accounting for the changes noted. The fear of COVID-19 infection from the hospital as well as other people in the hospital was cited as some of the reasons why they missed or delayed their vaccinations. This was worsened with the fact that the hospital had a treatment center for COVID-19. These reasons are supported by these quotes from some of the participants: “my child couldn't receive the first injection because I was afraid to come,” “People said all the COVID patients were being treated in the hospital,” and “I was really afraid that the staff were infected and they will subsequently infect myself and the child.”

Vaccine side effects were cited as another reason for the delay in bringing children for immunization, as stated by a participant in the statement: “As for me I delayed in bringing my child for immunization not because of the COVID but because of the side effects of the immunization that's why I delayed.”

Unavailability of vaccines and staff at the CWC was not cited as reason for the delay in the immunization. These was supported by quotes from two of the participants who stated that “We weren't told that the vaccines were not available because anytime we come and the injection is due, they inject the child” and “They (staff at the CWC) were always available.”

## 4. Discussion

This mixed study was carried out to ascertain the impact that the COVID-19 pandemic had on uptake of childhood vaccinations in the TTH by comparing the prepandemic coverage to the coverage in the pandemic era. Our study showed a massive drop in the average coverage of the vaccines. When individual vaccines were considered, a drop in coverage was seen in all but for the MR2 vaccine given at 18 months. The month of May 2020 was the most significantly affected month.

The indirect effect of the COVID-19 pandemic has been recorded in almost every aspect of healthcare: preventive and curative [9, 19]. Decline in pediatric hospital admissions has been reported as an indirect effect of the pandemic [7, 20]. Earlier studies documented another indirect effect of the pandemic on child health seen in decline in vaccinations [9, 15]. This decline is consistent to a report made in the Ebola pandemic which also had adverse indirect effects on healthcare delivery [16].

The decline in the vaccination coverage in our study ranged from 47.0 to 10.5%, depending on the vaccine. This drop was lower compared to a study by Buonsenso et al. who reported drops between 50 and 85% in Sierra Leone [21]. Our decline was however worse than the reported declines in the United Kingdom [14]. This disparity could be explained by the already existing immunization coverage in these countries, as immunization in countries with weaker health systems has been noted to be particularly affected by these disruptions such as this current pandemic [22, 23]. Although earlier studies had predicted that sub-Saharan Africa will be worst affected due to its weak health systems [18], this has not exactly been so [24]. However, the region has not been spared the indirect negative consequences of the pandemic [25].

The month of May 2020 recorded the highest decline in the vaccination schedule, as the country continued to see increased number of COVID-19 infections. With increasing infection and fear among the public, it was not surprising that we recorded such a massive decline. A WHO African regional analysis showed that the months of April 2020 and May 2020 recorded the highest declines from January to June 2020 [22].

The BCG vaccine given at birth was the most significantly affected vaccine. This may be due to a decline in the number of institutional deliveries or a decline in the number of births, with the former been more likely than the latter, as buttressed by previous studies [6, 26]. Also, we recorded lower declines with progress in the vaccine schedule. The MR2 vaccine did not even record a drop. This could possibly be due to the decrease in uptake of childhood vaccines with progress in vaccination schedule as noted in other studies [27, 28].

The negative indirect effect of the pandemic has been attributed to several factors. The fear of infection has been reported as a reason for delayed presentation of sick children to health facilities in the pandemic period even for curative services [23, 29]. This same reason was reported as one of the reasons accounting for the decline noted in our study. Other studies have supported this finding of ours [25, 30].

Closures of health facilities have also accounted for the delay or missing of vaccination schedule [23, 31]. A study in Morocco showed 22% of interviewed pediatricians completely halting their vaccination services [32]. We recorded decline in the uptake of childhood vaccinations, even though the childhood vaccination service was provided in the pandemic period, as similarly reported by other studies where services were being rendered despite the pandemic [33]. Mothers and staff interviewed also indicated that vaccination service was not closed down. However, it was worrying that such massive declines were observed.

Misinformation among the public is another factor reported to have accounted for the decline. This was seen by clients reporting that they heard vaccination has been halted due to the pandemic. Pandemic-related misinformation in the social media has been reported [34]. It is important that the public is educated well to understand the need for childhood vaccinations, despite the ongoing pandemic [35].

Lockdown measures instituted in some jurisdiction to curtail the spread of the COVID-19 virus have also been cited as a contributing factor for the decline in some areas [36]. However, Tamale was not affected by this measure that was implemented in some cities in the country [37].

The decline in vaccination noted in our study will need to be looked in a broader sense across the nation. This disruption is a potential source of outbreak of vaccine-preventable disease [25, 36].

Our study was not without limitations. It was a single-center study and may not reflect what was seen at other facilities that provide childhood vaccination services in the Tamale Metropolis. Unavailability of the data for December 2020 hindered us from making a direct comparison with this month of the previous year. Also, the limited number of caregivers interviewed in the focused group discussion, during our study period, might not have been representative of the entire caregivers who missed their children's immunization.

## 5. Conclusion and Recommendation

The COVID-19 pandemic has had a negative impact on childhood vaccination in the TTH. BCG given at birth was the most affected, among the vaccines studied. The fear of contracting the COVID-19 infection at the hospital was the commonly given reason for decline in uptake noted. There is an urgent need for a nationwide study to be conducted to examine the impact that the pandemic had on childhood vaccination in the country. Also, it is important that catch-up immunization schedule is instituted to curtail possible future outbreaks of vaccine-preventable diseases. In our setting, this can be achieved through mass callbacks using the media and professional societies in the Tamale Metropolis and direct phone calls to families. Through this means, defaulters will be enlightened on the importance of the vaccination, in prevention of vaccine-preventable diseases. Also, alternate means of vaccination can be instituted. For example, with the reopening of schools, daycare centers can be visited, so that children who might have missed certain vaccines would be given the doses [38].

## Figures and Tables

**Table 1:** Overall vaccinations done in the prepandemic era compared to the pandemic era.

Vaccines	Prepandemic era	Pandemic era	Change (%)
BCG	4891	2593	-2298 (-47.0)
PENTA 1	1921	1115	-806 (-42.0)
PENTA 2	1607	959	-648 (-40.3)
PENTA 3	1566	956	-610 (-39.0)
MR1	1102	986	-116 (-10.5)
MR2	580	585	5 (0.9)

**Table 2:** Monthly changes in vaccines administered.

Month	Prepandemic era	Pandemic era	Change (%)	Percentage delivered (prepandemic era)	Percentage delivered (pandemic era)
March	820	978	158 (19.3)	35.8	42.7
April	861	468	-393 (-45.6)	37.5	20.4
May	1152	339	-813 (-70.6)	50.2	14.8
June	1002	452	-550 (-54.9)	43.7	19.7
July	1090	520	-570 (-52.3)	47.5	22.7
August	1175	824	-351 (-29.8)	51.2	35.9
September	1316	868	-448 (-34.0)	57.4	37.9
October	1179	704	-475 (-40.3)	51.4	30.7
November	1304	816	-488 (-37.4)	56.9	35.6
January	1062	692	-370 (-34.8)	46.3	30.2
February	706	533	-170 (-24.5)	30.8	23.2
Total	11667	7194	-4473 (-38.3)		

## Data Availability

The dataset used to support findings of this study is available from the corresponding author on reasonable request.

## Supplementary Materials

Supplementary 1 Supplement 1: data collection sheet for uptake of vaccines used as proxy for the recommended age groups.

Supplementary 2 Supplement 2: in-depth discussion guide for the staff of the Child Welfare Clinic.

Supplementary 3 Supplementary 3: focused group discussion guide for caregivers who missed or delayed a vaccination schedule in the COVID-19 era.

## References

[1] Team EE (2020). Note from the editors: World Health Organization declares novel coronavirus (2019-nCoV) sixth public health emergency of international concern. *Eurosurveillance*.

[2] Wadvalla B.-A. (2020). How Africa has tackled covid-19. *BMJ*.

[3] Roberton T., Carter E. D., Chou V. B. (2020). Early estimates of the indirect effects of the COVID-19 pandemic on maternal and child mortality in low-income and middle-income countries: a modelling study. *The Lancet Global Health*.

[4] Hirabayashi K. (2020). *The impact of COVID-19 on the routine vaccinations: refletions during World Immunization Week*.

[5] Organization WH (2020). *At least 80 million children under one at risk of diseases such as diphtheria, measles and polio as COVID-19 disrupts routine vaccination efforts, warn Gavi, WHO and UNICEF*.

[6] Moyer C. A., Sakyi K. S., Sacks E., Compton S. D., Lori J. R., Williams J. E. (2021). COVID-19 is increasing Ghanaian pregnant women's anxiety and reducing healthcare seeking. *International Journal of Gynecology & Obstetrics*.

[7] Abdul-Mumin A., Cotache-Condor C., Bimpong K. A. (2021). Decrease in admissions and change in the diagnostic landscape in a newborn care unit in northern Ghana during the COVID-19 pandemic. *Frontiers in Pediatrics*.

[8] Abbas K., Procter S. R., van Zandvoort K. (2020). Routine childhood immunisation during the COVID-19 pandemic in Africa: a benefit-risk analysis of health benefits versus excess risk of SARS-CoV-2 infection. *The Lancet Global Health*.

[9] Bramer C. A., Kimmins L. M., Swanson R. (2020). Decline in child vaccination coverage during the COVID-19 pandemic—Michigan Care Improvement Registry, May 2016-May 2020. *American Journal of Transplantation*.

[10] Measles I. R. (2020). *More than 117 million children at risk of missing out on measles vaccine, as COVID-19 surges*.

[11] Dinleyici E. C., Borrow R., Safadi M. A. P., van Damme P., Munoz F. M. (2021). Vaccines and routine immunization strategies during the COVID-19 pandemic. *Human Vaccines & Immunotherapeutics*.

[12] Santoli J. M., Lindley M. C., DeSilva M. B. (2020). Effects of the COVID-19 pandemic on routine pediatric vaccine ordering and administration—United States, 2020. *MMWR Morbidity and Mortality Weekly Report*.

[13] Bramer C. A., Kimmins L. M., Swanson R. (2020). Decline in child vaccination coverage during the COVID-19 pandemic—Michigan Care Improvement Registry. *American Journal of Transplantation*.

[14] McDonald H. I., Tessier E., White J. M. (2020). Early impact of the coronavirus disease (COVID-19) pandemic and physical distancing measures on routine childhood vaccinations in England, January to April 2020. *Eurosurveillance*.

[15] Chandir S., Siddiqi D. A., Mehmood M. (2020). Impact of COVID-19 pandemic response on uptake of routine immunizations in Sindh, Pakistan: an analysis of provincial electronic immunization registry data. *Vaccine*.

[16] Camara B., Delamou A., Diro E. (2017). Influence of the 2014–2015 Ebola outbreak on the vaccination of children in a rural district of Guinea. *Public Health Action*.

[17] WHO Expanded Programme on Immunization. http://www.emro.who.int/pak/programmes/expanded-programme-on-immunization.html.

[18] Coker M., Folayan M. O., Michelow I. C., Oladokun R. E., Torbunde N., Sam-Agudu N. A. (2021). Things must not fall apart: the ripple effects of the COVID-19 pandemic on children in sub-Saharan Africa. *Pediatric Research*.

[19] Zhao J., Li H., Kung D., Fisher M., Shen Y., Liu R. (2020). Impact of the COVID-19 epidemic on stroke care and potential solutions. *Stroke*.

[20] Liguoro I., Pilotto C., Vergine M., Pusiol A., Vidal E., Cogo P. (2021). The impact of COVID-19 on a tertiary care pediatric emergency department. *European Journal of Pediatrics*.

[21] Buonsenso D., Cinicola B., Kallon M. N., Iodice F. (2020). Child healthcare and immunizations in sub-Saharan Africa during the COVID-19 pandemic. *Frontiers in Pediatrics*.

[22] Masresha B. G., Luce Jr R., Shibeshi M. E. (2020). The performance of routine immunization in selected African countries during the first six months of the COVID-19 pandemic. *The Pan African Medical Journal*.

[23] Chanchlani N., Buchanan F., Gill P. J. (2020). Addressing the indirect effects of COVID-19 on the health of children and young people. *CMAJ*.

[24] Causey K., Fullman N., Sorensen R. J. (2021). Estimating global and regional disruptions to routine childhood vaccine coverage during the COVID-19 pandemic in 2020: a modelling study. *The Lancet*.

[25] Lassi Z. S., Naseem R., Salam R. A., Siddiqui F., Das J. K. (2021). The impact of the COVID-19 pandemic on immunization campaigns and programs: a systematic review. *International Journal of Environmental Research and Public Health*.

[26] Ashish K., Peterson S. S., Gurung R. (2021). The perfect storm: disruptions to institutional delivery care arising from the COVID-19 pandemic in Nepal. *Globalization and Health*.

[27] the PROMISE-EBF Study Group, Fadnes L. T., Jackson D. (2011). Vaccination coverage and timeliness in three South African areas: a prospective study. *BMC Public Health*.

[28] Laryea D. O., Abbeyquaye Parbie E., Frimpong E. (2014). Timeliness of childhood vaccine uptake among children attending a tertiary health service facility-based immunisation clinic in Ghana. *BMC Public Health*.

[29] Kirmani S., Saleem A. (2021). Impact of COVID-19 pandemic on paediatric services at a referral centre in Pakistan: lessons from a low-income and middle-income country setting. *Archives of Disease in Childhood*.

[30] Bechini A., Garamella G., Giammarco B. (2020). Paediatric activities and adherence to vaccinations during the COVID-19 epidemic period in Tuscany, Italy: a survey of paediatricians. *Journal of Preventive Medicine and Hygiene*.

[31] WHO At least 80 million children under one at risk of diseases such as diphtheria, measles and polio as COVID-19 disrupts routine vaccination efforts, warn Gavi, WHO and UNICEF 2020. http://www.who.int/news/item/22-05-2020-at-least-80-million-children-under-one-at-risk-of-diseases-such-as-diphtheria-measles-and-polio-as-covid-19-disrupts-routine-vaccination-efforts-warn-gavi-who-and-unicef.

[32] Chekhlabi N., Arrab R., Ettair S., Dini N. (2021). Effects of the COVID-19 pandemic on childhood immunization in Morocco: electronic survey of 103 pediatricians. *The Pan African Medical Journal*.

[33] Vogt T. M., Zhang F., Banks M. (2020). Provision of pediatric immunization services during the COVID-19 pandemic: an assessment of capacity among pediatric immunization providers participating in the Vaccines for Children program—United States, May 2020. *Morbidity and Mortality Weekly Report*.

[34] Chen K., Luo Y., Hu A., Zhao J., Zhang L. (2021). Characteristics of misinformation spreading on social media during the COVID-19 outbreak in China: a descriptive analysis. *Risk Management and Healthcare Policy*.

[35] Zhong Y., Clapham H. E., Aishworiya R. (2021). Childhood vaccinations: hidden impact of COVID-19 on children in Singapore. *Vaccine*.

[36] Chandir S., Siddiqi D. A., Setayesh H., Khan A. J. (2020). Impact of COVID-19 lockdown on routine immunisation in Karachi, Pakistan. *The Lancet Global Health*.

[37] Acheampong K. (2020). Covid-19: Accra, Kumasi empty as lockdown takes effect. https://starrfm.com.gh/2020/03/covid-19-accra-kumasi-empty-as-lockdown-takes-effect/.

[38] WHO Closing immunization gaps caused by COVID-19. https://www.who.int/immunization/programmes_systems/policies_strategies/Closing_Immunization_Gaps_caused_by_COVID-19_v11.pdf.

