# Supplementary material for: Decline in Uptake of Childhood Vaccinations in a Tertiary Hospital in Northern Ghana during the COVID-19 Pandemic
Source: Biomed Res Int. 2021 Dec 14;2021:6995096. doi: 10.1155/2021/6995096 (PMC8672106; doi:10.1155/2021/6995096)
Supplement: Supplementary 1 — Supplement 1: data collection sheet for uptake of vaccines used as proxy for the recommended age groups. [file 6995096.f1.docx]

**Supplement 1: Data collection sheet** **for uptake of vaccines used as proxy for the recommended age groups.**

| **Month** | **BCG** | **Penta 1** | **Penta 2** | **Penta 3** | **MR1** | **MR2** |
| --- | --- | --- | --- | --- | --- | --- |
| March, 2019 |  |  |  |  |  |  |
| April, 2019 |  |  |  |  |  |  |
| May, 2019 |  |  |  |  |  |  |
| June, 2019 |  |  |  |  |  |  |
| July, 2019 |  |  |  |  |  |  |
| August, 2019 |  |  |  |  |  |  |
| September, 2019 |  |  |  |  |  |  |
| October, 2019 |  |  |  |  |  |  |
| November, 2019 |  |  |  |  |  |  |
| December, 2019 |  |  |  |  |  |  |
| January, 2020 |  |  |  |  |  |  |
| February, 2020 |  |  |  |  |  |  |
| March, 2020 |  |  |  |  |  |  |
| April, 2020 |  |  |  |  |  |  |
| May, 2020 |  |  |  |  |  |  |
| June, 2020 |  |  |  |  |  |  |
| July, 2020 |  |  |  |  |  |  |
| August, 2020 |  |  |  |  |  |  |
| September, 2020 |  |  |  |  |  |  |
| October, 2020 |  |  |  |  |  |  |
| November, 2020 |  |  |  |  |  |  |
| December, 2020 |  |  |  |  |  |  |
| January, 2021 |  |  |  |  |  |  |
| February, 2021 |  |  |  |  |  |  |
