# Supplementary material for: Decline in Uptake of Childhood Vaccinations in a Tertiary Hospital in Northern Ghana during the COVID-19 Pandemic
Source: Biomed Res Int. 2021 Dec 14;2021:6995096. doi: 10.1155/2021/6995096 (PMC8672106; doi:10.1155/2021/6995096)
Supplement: Supplementary 2 — Supplement 2: in-depth discussion guide for the staff of the Child Welfare Clinic. [file 6995096.f2.doc]

**Supplement 2: In-dept discussion Guide for Staff of the Child Welfare Clinic**

First, we appreciate the time you have made for this meeting. This in-dept interview is to understand the impact of the COVID-19 pandemic on childhood vaccination in the Tamale Teaching Hospital. Please base what you say on what you have observed.

The recordings for this study will be anonymous. We don’t want you to say your name at the start, but we have given each of you a pseudonym (made up name), and will go around in turn asking you to say your pseudonym, what age you are, the area in which you live, and what your job is. This will help us to identify individual voices when we come to write up the interviews.

The study is optional, please. You can opt-out at any point in time during the session. We will like to seek your consent also for this interview.

We would your perspective on two main topics: Change in immunization schedule in the covid-19 era and possible reasons for the change.

**Change in immunization schedule in the covid-19**

Firstly, we wish to know if you have observed any change in the vaccination schedule during the covid-19 era?

**Possible reasons do you think accounted for this change**

Prompts, if necessary

For example:

- Fear of patient’s caregivers getting infected with the Virus?
- Availability of a vaccine?
- Healthcare workers asked caregivers not to come to the hospital?
- Shift system to curtail spread of virus
- Reassignment of public health workers and to contact tracing?
- Reassignment of public health nurse to covid treatment and isolation centers?
- Understaffing because of infection among health workers?
- Directive for superiors or hospital management?
- Absence of clear policy guidelines?
- Fear of getting infected?

Please do you have any questions for us?

Thank you

Name:

Signature:
