# Supplementary material for: Decline in Uptake of Childhood Vaccinations in a Tertiary Hospital in Northern Ghana during the COVID-19 Pandemic
Source: Biomed Res Int. 2021 Dec 14;2021:6995096. doi: 10.1155/2021/6995096 (PMC8672106; doi:10.1155/2021/6995096)
Supplement: Supplementary 3 — focused group discussion guide for caregivers who missed or delayed a vaccination schedule in the COVID-19 era. [file 6995096.f3.docx]

**Supplement 2: In-dept discussion Guide for caregivers who missed or delayed a vaccination schedule in the COVID-19 era.**

First, we appreciate the time you have made for this meeting. This focused group discussion is to understand the impact of COVID-19 pandemic on childhood vaccination in the Tamale Teaching Hospital.

The recordings for this study will be anonymous. We don’t want you to say your name at the start, but we have given each of you a pseudonym (made up name), and will go around in turn asking you to say your pseudonym, what age you are, the area in which you live, and what your job is. This will help us to identify individual voices when we come to write up the interviews.

The study is optional, please. You can opt-out at any point in time during the session. We will like to seek your consent also for this interview.

We will like to seek your consent also for this group discussion.

We would your perspective on reasons(s) why you missed out on the schedule.

**Possible reasons do you think accounted for this change**

Prompts, if necessary

For example:

- Fear of caregiver getting infected with covid-19?
- Fear of exposure of babies to infection with covid-19?
- Availability of a vaccine?
- Healthcare workers asked you not to come to the hospital?
- Healthcare workers were not available.

Does anyone have any questions for us?

Thank-you

Name:

Signature:
